# Over-expression of *Slc30a8*/ZnT8 selectively in the mouse α cell impairs glucagon release and responses to hypoglycemia

**DOI:** 10.1186/s12986-016-0104-z

**Published:** 2016-07-07

**Authors:** Antonia Solomou, Erwann Philippe, Pauline Chabosseau, Stephanie Migrenne-Li, Julien Gaitan, Jochen Lang, Christophe Magnan, Guy A. Rutter

**Affiliations:** Section of Cell Biology and Functional Genomics, Division of Diabetes Endocrinology and Metabolism, Department of Medicine, Imperial College London, Imperial Centre for Translational and Experimental Medicine, Hammersmith Hospital, du Cane Road, London, W12 0NN UK; University Paris Diderot-Paris 7-Unit of Functional and Adaptive Biology (BFA) UMR 8251 CNRS, Paris, France; CNRS UMR 5248, Chimie et Biologie des Membranes et Nano-objets, Université de Bordeaux, F-33615 Pessac, France

## Abstract

**Background:**

The human *SLC30A8* gene encodes the secretory granule-localised zinc transporter ZnT8 whose expression is chiefly restricted to the endocrine pancreas. Single nucleotide polymorphisms (SNPs) in the human *SLC30A8* gene have been associated, through genome-wide studies, with altered type 2 diabetes risk. In addition to a role in the control of insulin release, recent studies involving targeted gene ablation from the pancreatic α cell (Solomou et al., *J Biol Chem* 290(35):21432-42) have also implicated ZnT8 in the control of glucagon release. Up to now, however, the possibility that increased levels of the transporter in these cells may impact glucagon secretion has not been explored.

**Methods:**

Here, we use a recently-developed reverse tetracyline transactivator promoter-regulated ZnT8 transgene to drive the over-expression of human ZnT8 selectively in the α cell in adult mice. Glucose homeostasis and glucagon secretion were subsequently assessed both in vivo during hypoglycemic clamps and from isolated islets in vitro.

**Results:**

Doxyclin-dependent human ZnT8 mRNA expression was apparent in both isolated islets and in fluorescence-activated cell sorting- (FACS) purified α cells. Examined at 12 weeks of age, intraperitoneal glucose (1 g/kg) tolerance was unchanged in transgenic mice versus wild-type littermates (*n* = 8-10 mice/genotype, *p* > 0.05) and sensitivity to intraperitoneal insulin (0.75U/kg) was similarly unaltered in transgenic animals. In contrast, under hyperinsulinemic-hypoglycemic clamp, a ~45 % (*p* < 0.001) reduction in glucose infusion rate was apparent, and glucagon release was significantly (~40 %, *p* < 0.01) impaired, in transgenic mice. Correspondingly, examined in vitro, glucagon secretion was significantly reduced (~30 %, *p* < 0.05) from transgenic *versus* control islets at low, stimulatory glucose concentrations (1 mM, *p* < 0.05) but not at high glucose (17 mM) glucose (*p* > 0.05). Over-expression of ZnT8 in glucagonoma-derived αTC1-9 cells increased granule free Zn^2+^ concentrations consistent with a role for Zn^2+^ in this compartment in the action of ZnT8 on glucagon secretion.

**Conclusions:**

Increased ZnT8 expression, and a likely increase in intragranular free Zn^2+^ concentration, is deleterious in pancreatic α cells for stimulated glucagon release. These data provide further evidence that type 2 diabetes-associated polymorphisms in the *SLC30A8*/ZnT8 gene may act in part via alterations in glucagon release and suggest that ZnT8 activation may restrict glucagon release in some settings.

## Background

Type 2 diabetes mellitus (T2D) is a complex polygenic disease which affects ~ 1 in 12 of the adult population and consumes ~10 % of the health care budgets of most westernized societies [1]. Although genome-wide association (GWA) and other studies have in recent years identified multiple loci as affecting the risk T2D [2, 3], functional work in model systems remains important if we are fully to understand the physiological role(s), and potential as pharmacotherapeutic targets, of the implicated genes. Most such studies up to now have used gene deletion in mice, inactivating candidates either globally or in disease-relevant tissues [4, 5]. In the case of the endocrine pancreas-restricted secretory granule zinc transporter *SLC30A8*/ZnT8, identified in GWA studies for T2D [6], this approach has been pivotal in highlighting the role of the transporter in the control of secretory granule formation and Zn^2+^ storage [7, 8] insulin secretion [9] and hepatic clearance of the hormone [10].

Nevertheless, the impact of *SLC30A8* risk variants [2] on ZnT8 activity and T2D risk are still debated. The common risk variant rs13266634 in the *SLC30A8* gene encodes an amino acid exchange (R325W) which is believed to lower transporter activity [7, 11]. On the other hand, rare truncating variants of ZnT8 are protective [12]. The reasons for this complex relationship between ZnT8 levels and disease risk are not fully understood [13, 14]. Whilst the role of the transporter in the control of insulin secretion has been the chief focus of interest in recent years, the observation that ZnT8 is also expressed in the α cell in both rodents [7] and humans [15] leads to the possibility that an action via glucagon release may also affect diabetes risk. Indeed, Zn^2+^ ions have been shown by autometallography [16] to be present in the secretory granule of α as well as β cells. Correspondingly, we have recently shown, by α cell-selective deletion of ZnT8 in mice [17], an important role for this transporter in the control of glucagon secretion.

Importantly, and as well as providing insights into the aetiopathology of T2D, changes in the normal release of glucagon may also have consequences for glycemic control in Type 1 diabetes (T1D). In the latter disease, inadequate responses to hypoglycaemia constitute a substantial risk and limit the use of insulin treatment to achieve good glycemic control and minimize disease complications [18].

Although investigating the impact of the absence of a gene is usually highly informative, its overexpression may also provide important insights, particularly with respect to the possible impact of pharmacological approaches which activate the gene or its product. Inducible expression systems are consequently often used in mice to achieve both temporal and spatial (i.e. tissue-specific) control of the expression of a given gene. Components of the Tet Switches [19] originate from the tetracycline (Tet) resistance operon in *E.coli* and belong to one of the most evolved gene regulation systems. “Tet-Off” and “Tet-On” systems are used in the majority of the studies involving inducible expression. The Tet-Off system was initially developed in 1992 and in the presence of the antibiotic tetracycline the expression from a Tet-inducible promoter is decreased [19].

In order to utilize tetracycline as a regulator of transcription, a tetracycline-controlled transactivator (tTA) is controlled by fusion of the tetracycline repressor with a transcriptional activation domain from Herpes Simplex Virus (HSV). Thus, in the absence of tetracycline, the fusion protein can bind *tet* operator sequences and promote transcription while in the presence of the antibiotic, its binding to the protein makes it unable to bind DNA leading to a decrease in gene expression. The “Tet-On” system was later developed by mutation of the repressor portion of the tTA to create a reverse tetracycline controlled transactivator (rtTA) that relies on tetracycline for induction of gene expression rather than repression [20]. The system was first used in the pancreatic β-cell by Efrat and colleagues [21] and about ten years later in the α-cell [22].

Recently, our laboratory used this approach to determine the effects of ZnT8 over-expression in the pancreatic β-cell in mice, driving rtTA expression with the rat insulin 2 promoter [23]. In the present study, the rtTA sequence was placed under the control of the preproglucagon promoter in Glu-rtTA mice [22] allowing us to drive the expression of ZnT8 selectively in the α-cell in the adult mouse.

Using this approach we have investigated the effect of ZnT8 overexpression on glucagon secretion. Glu-rtTA mice were therefore crossed to mice bearing a human ZnT8 transgene whose expression was driven by the *tet* operator sequence. In contrast to the recently described effect of α cell-selective deletion of ZnT8 to enhance glucagon secretion at low glucose [17], we demonstrate that ZnT8 over-expression results in the suppression of glucagon release during hypoglycaemia, consequently enhancing glucose clearance.

## Methods

### Materials

Chemicals and biochemical were purchased from Sigma-Aldrich (Poole, Dorset, U.K.) unless otherwise indicated.

### Generation and genotyping of αZnT8Tg mice

Glu-rtTA mice, which possess a 1.1 kb region of the preproglucagon promoter upstream of rtTA [22], were crossed to animals bearing a human ZnT8-overexpressing transgene (ZnT8Tg) as described in [23]. Heterozygous Glu-rtTA mice were crossed to homozygous ZnT8Tg animals. Two ZnT8Tg founders were used, corresponding to lines #31 and #23 in [23]. The litters comprised Glu-rtTA offspring, which expressed the human ZnT8 transgene after induction with docycycline (as described below), while littermates bearing the ZnT8Tg allele alone, and born at Mendelian frequency (50 %), were used as controls. Doxycyclin, a tetracycline derivative, was given to all experimental mice in the drinking water (2 g/L) from the age of six weeks in order to induce ZnT8 expression in Glu-rtTA^+^:ZnT8Tg^+^ animals [22]. Animals were kept in a pathogen-free facility under a 12 h light-dark cycle with access to water and a standard mouse diet (Lillico Biotechnology). After weaning at 3–4 weeks of age mice were housed two to five per cage. All strains were maintained on a C57/BL6/J background.

**Fig. 1:**
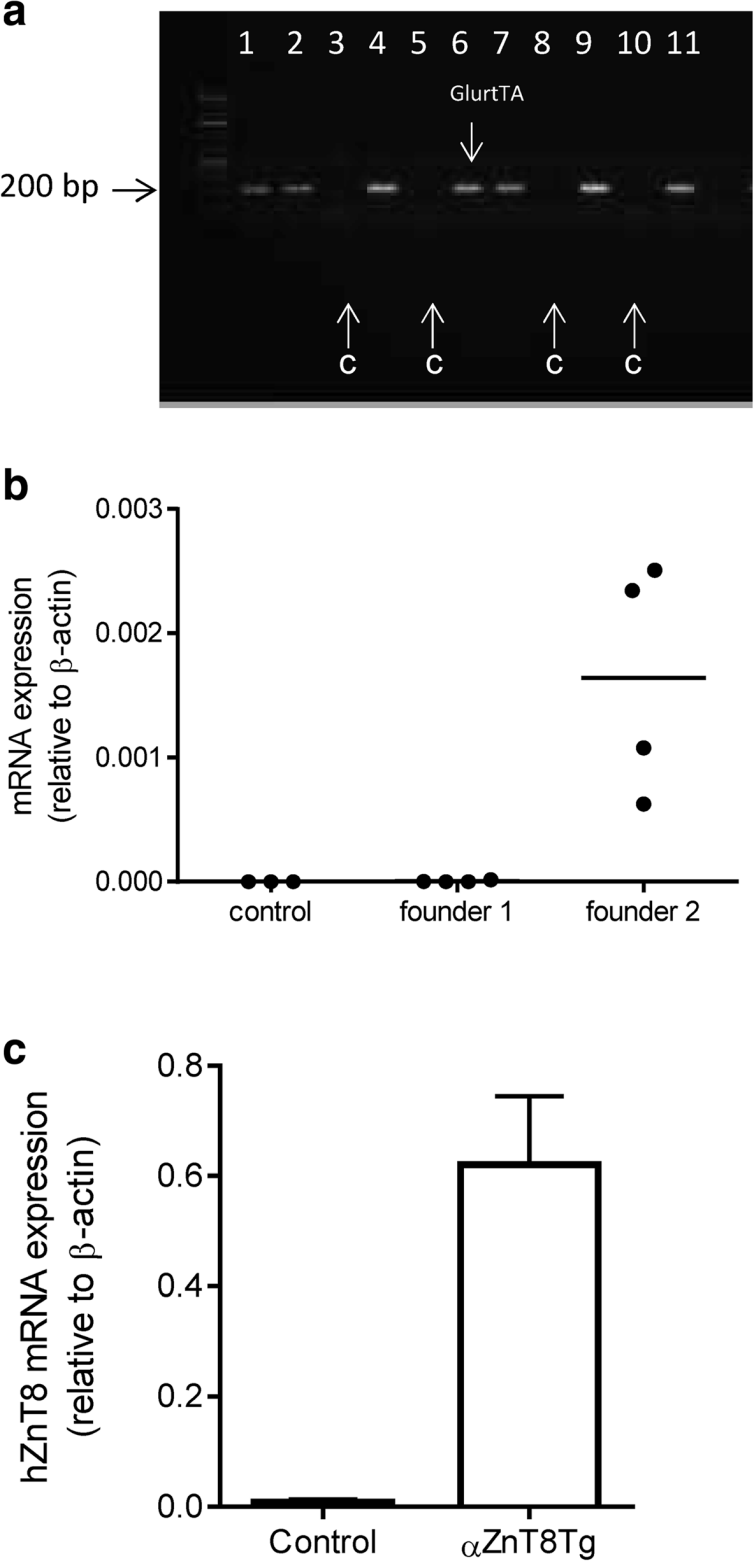
Overexpression of human ZnT8 in the α cell. **a** Genotyping by PCR. The Glu-rtTA transgene was amplified by standard PCR to identify control and transgenic animals. Lanes derived from control (lacking the GlrTTA transgene) mice are indicated with arrows as “C”. Samples from transgene-bearing mice are present in lanes 1,2,4,6,7,9,11. **b** Human ZnT8 expression in isolated islets. Total RNA was extracted from islets from control and transgenic mice. The levels of hZnT8 were assessed by RT-qPCR. **c** Human ZnT8 expression in purified α-cells. RNA was extracted from α-cells obtained from control or αZnT8Tg mice. The levels of hZnT8 mRNA were assessed by RT-qPCR

Genotyping for Glu-rtTA was performed using standard PCR on DNA extracted from ear biopsies (Fig. 1a) using the primers indicated in Table 1. For the ZnT8 transgene, qPCR was used to detect the presence in the genome of the firefly luciferase gene, included in the transgene in the antisense direction with respect to ZnT8 cDNA [23].

**Table 1:** PCR primers

Gene	Sequence
β Actin	FOR: CGAGTCGCGTCCACCC
	REV: CATCCATGGCGAACTGGTG
Glucagon	FOR: CCAAGAGGAACCGGAACAAC
	REV: CCTTCAGCATGCCTCTCAAAT
Human ZnT8	FOR: CTGTCATCGAAGCCTCCCTC
	REV: AAGGGCATGCACAAAAGCAG
Glu-rtTA	FOR: CATAAACGGCGCTCTGGAATTACTCAATGGAGTCG
	REV: GCCGAGATGCACTTTAGCCCCGTCGCGATGTGAGA
Luciferase	FOR: CAACTGCATAAGGCTATGAAGAGA
	REV: ATTTGTATTCAGCCCATATCGTTT

### Islet isolation and culture

Mice were sacrificed by cervical dislocation and islets isolated essentially as described [24]. In brief, pancreata were inflated with collagenase solution at 1ug/ml (Serva) and placed in a water bath at 37 °C for 10 min. After centrifugation and washing islets were purified on a Histopaque 119 (Sigma) gradient, by centrifugation at 2500 rpm for 20 min. The islet layer was recovered and islets cultured in complete RPMI 1640 medium (Gibco; 11 mM glucose) for 2–4 h at 37 °C islets before hand-picking into fresh media.

### Islet dissociation, fluorescence-activated cell sorting (FACS) and FACS analysis

FACS analysis was performed essentially as described [17]. After overnight incubation, 250–300 islets of the same genotype were handpicked and dissociated into single cells by repeated pipetting in 150 μl of Hank’s based cell dissociation buffer (Invitrogen) containing 0.1 % BSA and 0.1 % trypsin. The reaction was stopped with the addition of FBS (20 μl; Seralab). Cells were incubated in near-IR dead cell stain (Life technologies) for 20 min. at 4 °C, washed with PBA (PBS, 1 % BSA, 0.1 % azide) and fixed in 2 % (w/v) PFA for 10 min. and then washed twice with PBA and once with Saponin (0.025 % in PBA) before 10 min incubation with Saponin at room temperature. Cells were incubated with primary antibodies against insulin or glucagon (see Table 2) before incubation with secondary antibodies (anti-mouse AF 405, anti-guinea pig AF 488, anti-rabbit AF 640) for 20 min and resuspension in PBA. The samples were run on a BD Fortessa Flow Cytometer (BD Bioscience).

**Table 2:** Antibodies

Primary			
Antibody	Species	Company	Dilution
ZnT8	Rabbit	Mellitech	1:200
Glucagon	Mouse	Sigma	1:1000
Glucagon	Rabbit	Santa Cruz	1:200
Insulin	Guinea pig	Santa Cruz	1:200
Secondary			
Antibody	Species	Company	Dilution
Anti-mouse Alexa 568	Goat	Invitrogen	1:1000
Anti-rabbit Alexa 488	Goat	Invitrogen	1:1000
Anti-guinea pig Alexa 488	Donkey	Invitrogen	1:1000
Anti-mouse Alexa 488	Donkey	Invitrogen	1:1000
Anti-rabbit 568	Goat	Invitrogen	1:1000

### RNA extraction and cDNA synthesis

Islets were washed once in phosphate-buffered saline (PBS) followed by addition of TRIzol (ThermoFisher). Chloroform (200 μl per ml of TRIzol) was added and after centrifugation the upper aqueous phase was removed and RNA was precipitated by adding 400 μl isopropanol per ml of TRIzol. After re-centrifugation and washing the dried pellet was finally resuspended in nuclease-free water. The same quantity of RNA was used from each sample to perform a reverse transcription polymerase chain reaction (RT-PCR) and cDNA synthesis using random primers. cDNA was generated from RNA (up to 1 μg) using a High Capacity Reverse Transcription kit (Applied Biosystems) according the manufacturer’s instructions using the following thermocycler protocol: 10 min 25 °C, 2 h 37 °C, 5 min. 85 °C, hold 4 °C.

### Quantitative PCR (qPCR)

For gene expression measurements cDNA (2 μl) from the RT-PCR reaction above were used as template for quantitative Real Time PCR (qPCR) using a Fast SYBR Green Master Mix (Invitrogen) according to the manufacturer’s instructions. The reaction was initiated at 50 °C for 2 min. followed by the activation and pre-denaturation step at 95 °C for 10 min. The run was made up of 40 cycles of 15 s at 95 °C and 1 min. at 60 °C.

### αTC1.9 cell transfection

αTC1.9 cells were transfected with a human ZnT8 (W325 variant) expression vector or with the corresponding empty vector (EV), using Lipofectamine2000 (LifeTechnologies) as previously described [25, 26] and according to the manufacturer’s instruction. For cytosolic Zn^2+^ measurement, cells were co-transfected with an eCALWY-4 [27] expression vector. Imaging experiments were performed the day after transfection.

### Cytosolic Zn^2+^ measurement with eCALWY-4

Cells on coverslips were washed twice in Krebs-HEPES-bicarbonate (KHB) buffer (140 mM NaCl, 3.6 mM KCl, 0.5 mM NaH_2_PO_4_, 0.2 mM MgSO_4_, 1.5 mM CaCl_2_, 10 mM HEPES, 25 mM NaHCO_3_), which was warmed, bubbled with 95:5 O_2_/CO_2_, set to pH 7.4, and contained 11 mM glucose. Imaging of [Zn^2+^] using eCALWY sensors [27] was carried out as optimized before [25, 26]. Briefly, cells were maintained at 37 °C throughout with a heating stage (MC60, LINKAM, Scientific Instruments), and KHB was perifused (1.5 to 2 mL/min) with additions as stated in the figures. Images were captured at 433 nm monochromatic excitation wavelength (Polychrome IV, Till photonics) using an Olympus IX-70 wide-field microscope with a 40×/1.35NA oil immersion objective and a zyla sCMOS camera (Andor Technology) controlled by Micromanager software. Acquisition rate was 20 images/min. Emitted light was splitted and filtered by a Dual-View beam splitter (Photometrics) equipped with a 505dcxn dichroic mirror and two emission filters (Chroma Technology, D470/24 for cerulean and D535/30 for citrine). Image analysis was performed with ImageJ software using a homemade macro and the fluorescence emission ratios were derived after subtracting background. Steady-state fluorescence intensity ratio citrine/cerulean (R) was measured, then maximum and minimum ratios were determined to calculate free Zn^2+^ concentration using the following formula: [Zn^2+^] = *K*_d_(R_max_ – R)/(R – R_min_). The maximum ratio (R_max_) was obtained upon intracellular zinc chelation with 50 μM TPEN and the minimum ratio (R_min_) was obtain upon zinc saturation with 100 μM ZnCl_2_ in the presence of the Zn^2+^ ionophore, pyrithione (5 μM).

### Granular Zn^2+^ Imaging with Zinpyr-4

αTC1.9 cells were incubated for 20 min. before imaging in 1 μM Zinpyr-4 (Santa Cruz Biotechnology), and washed twice in imaging buffer. Acquisitions were performed as described above with additions as stated on the figures. Zinpyr-4 was excited at 488 nm wavelength and signal was collected through a 535/50 nm emission filter. Differences in Zinpyr4 mean intensity between EV and ZnT8-expressing cells were measured using ImageJ.

### Other methods

Glucagon secretion was measured from islets essentially as described in detail in [17]. In brief, 18 size-matched islets per condition were incubated with constant agitation for 1 h in 0.5 mL Krebs-HEPES bicarbonate buffer at 37 °C with either 1 mM or 17 mM glucose, and total and secreted glucagon measured at the end of the incubations by radioimmunoassay (Millipore). Details of intraperitoneal glucose (1 g/kg; IPGTT) and insulin (0.5 U/kg; IPITT) tolerance tests, and hypoglycemic clamps are provided in [17].

### Statistics

Student’s *t* test was used to identify differences between two independent variables and assessments between multiple variables assessed by two-way ANOVA (with suitable correction from multiple tests), followed by Bonferroni’s post hoc test. All analyses were performed using GraphPad Prism 6.0, and *p* < 0.05 was considered significant. Values are presented as means ± S.E.

## Results

### Expression of human ZnT8 in transgenic islets

Initially to check that the administration of doxycyclin was effective in inducing the overexpression of ZnT8, total RNA was extracted from isolated islets incubated overnight with doxycyclin (5 μg/ml). qPCR analysis revealed expression of the human form of ZnT8 in the mice containing both the overexpressing transgene and the Glu-rtTA but not in the control mice carrying only the ZnT8 transgene (Fig. 1b). We tested both founders that carried the transgene and observed that only founder 2 (#31 from [23]) expressed detectable levels of human ZnT8. Consequently, offspring from this founder was used in all in experiments.

In order to obtain a more accurate assessment of the degree of overexpression in a pure α-cell population and as opposed to islets, which are largely composed of β-cells [28], isolated islets were dissociated and stained with glucagon for fluorescence-activated cell sorting (FACS). RNA was then extracted from the sorted cells to check for expression of the human ZnT8 in the α-cells obtained from transgenic and control animals (Fig. 1c). Whilst essentially absent from non-transgenic islets, hZnT8 mRNA was readily detected in islets from transgenic animals.

### Intraperitoneal glucose and insulin tolerance are unaltered in αZnT8Tg mice

**Fig. 2:**
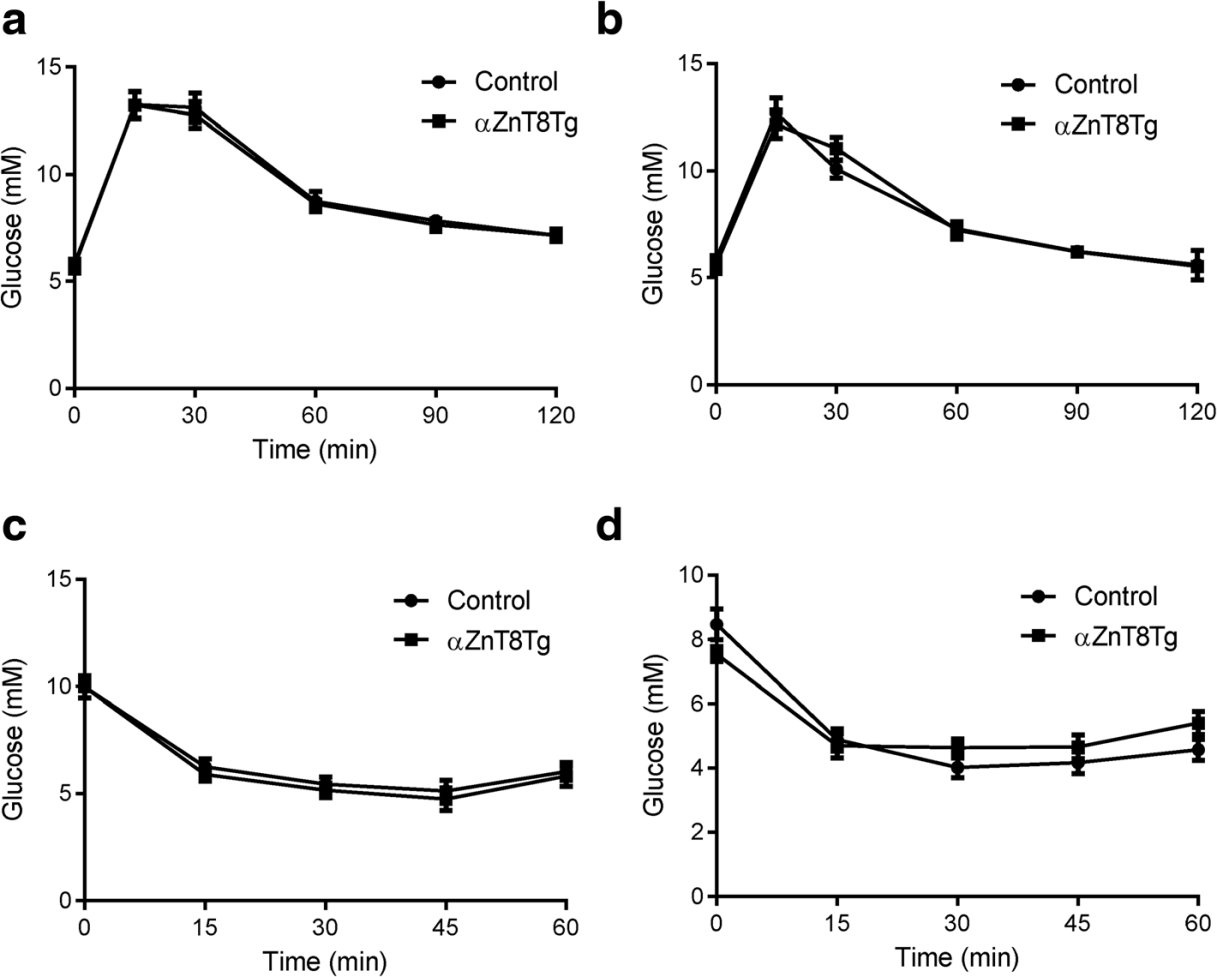
Glucose and insulin tolerance in αZnT8Tg mice. Intraperitoneal glucose tolerance tests were performed on control and transgenic male (**a**) and female (**b**) mice at 8 weeks of age. *n* = 8–10 mice/genotype. Animals were fasted for 16 h before injection of 1 g/kg glucose. Blood glucose was sampled after venisection of the tail vein at the indicated times, and quantified using an automated glucometer (AccuCheck, Roche). **c**, **d** Insulin sensitivity. Intraperitoneal insulin tolerance tests performed on 12 week old male (**c**) and female (**d**) mice fasted for 5 h and injected with 0.5U/kg insulin. *n* = 9–10 mice per genotype. Blood sampling was performed as in (**a**, **b**)

To assess whether glucose homeostasis in transgenic mice was altered, intraperitoneal glucose tolerance tests (IPGTTs) were performed on male (Fig. 2a) and female (Fig. 2b) mice aged 8 weeks. In neither case were any differences in the excursions in blood glucose observed between control and transgenic animals at any time point during the tests.

Insulin sensitivity was similarly determine by intraperitoneal insulin tolerance tests (ITTs) were performed on transgenic and control male (Fig. 2c) and female (Fig. 2d) mice at the age of 14 weeks. Again, no differences were observed between the responses of αZnT8Tg and control mice.

### Trangenic mice require an elevated glucose infusion rate (GIR) and display impaired glucagon secretion during hypoglycemic clamps

**Fig. 3:**
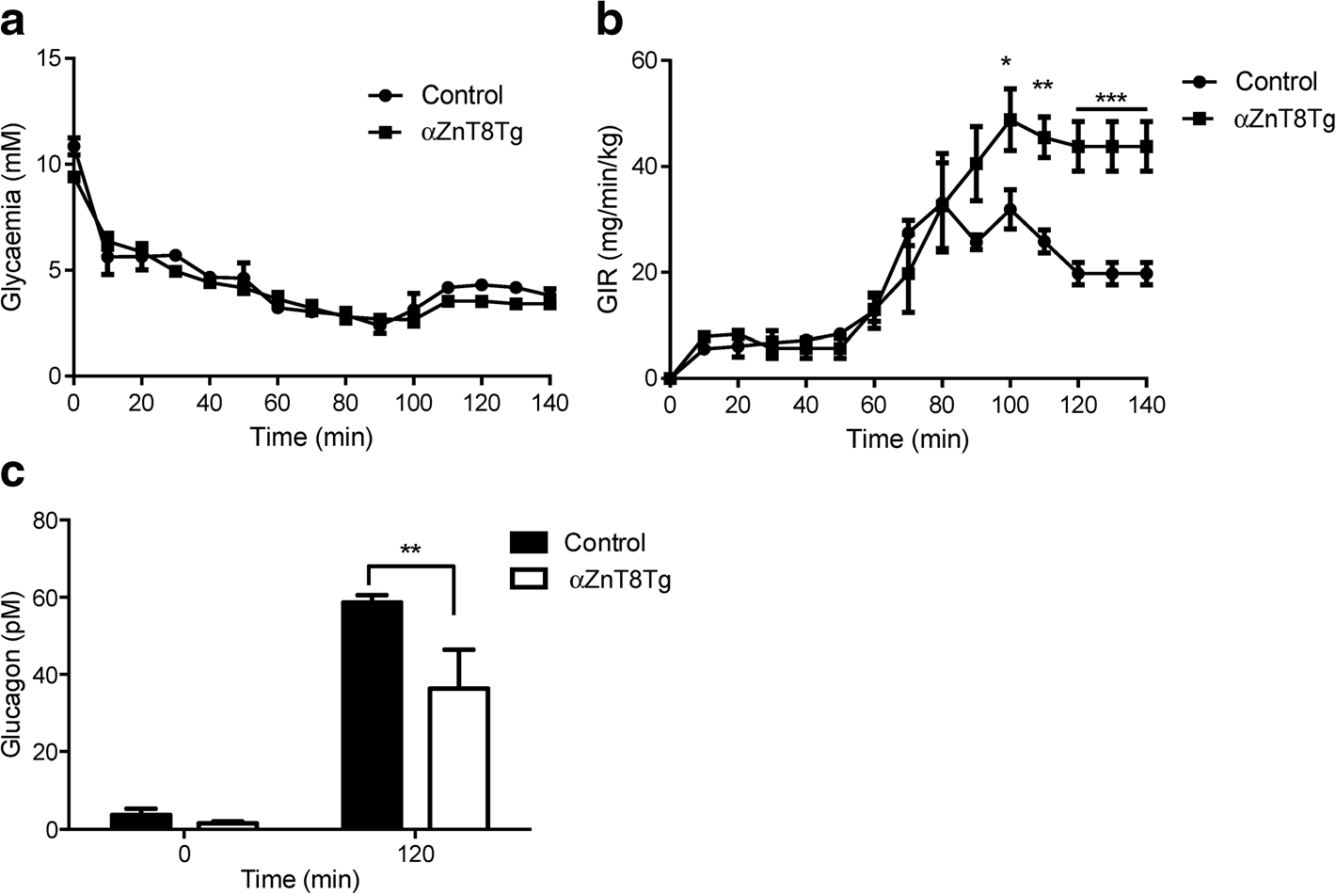
Elevated glucose infusion rates in αZnT8 KO mice during hypoglycaemic hyperinsulinaemic clamps. Experiments were performed on 12 week old male mice. Animals were fasted for 4 h and rendered hypoglycaemic by infusion of insulin (**a**). Glucose levels were monitored and glucose infusion rate adjusted accordingly to keep the mice in a hypoglycaemic state at ~60–70 % of the initial value (**b**). **c** Blood was collected from tail vein at times 0 and 120 min in tubes filled with EGTA (1.6 mg/ml, Sigma) and aprotinin (250 k-international units/ml; Sigma) for glucagon measurement..*n* = 4 mice per genotype. Data are mean ± SEM, *P < 0.05, **P < 0.01, ***P < 0.005. Other details are given in the Methods section

ITTs as described above could only be used safely (i.e. without life-threatening hypoglycaemia) to provide a limited decrease in blood glucose levels (to ~ 4 mM), above the usual threshold for substantial glucagon release in the mouse [29]. In order to elicit a more marked, but stable, decrease in blood glucose levels, where glucagon release is likely to be more strongly stimulated, hypoglycaemic clamps [17] were performed. Male mice were thus rendered hypoglycaemic by continuous intravenous administration of insulin (Fig. 3a). Under these conditions ZnT8-overexpressing mice required a significantly higher glucose infusion rate (GIR) to maintain hypoglycaemia compared to control mice (Fig. 3b), suggesting an impairment in glucagon release. Plasma glucagon levels were consequently measured prior to the onset of hypoglycaemia and at 120 min. after the beginning of insulin infusion (Fig. 3c). Whilst no differences were observed between glucagon levels in control and transgenic mice at the earlier time point, concentrations of the hormone at 120 min were ~45 % lower (*p* < 0.01) in transgenic versus control mice (Fig. 3c).

### Glucagon secretion is impaired in islets isolated from αZnT8Tg mice

**Fig. 4:**
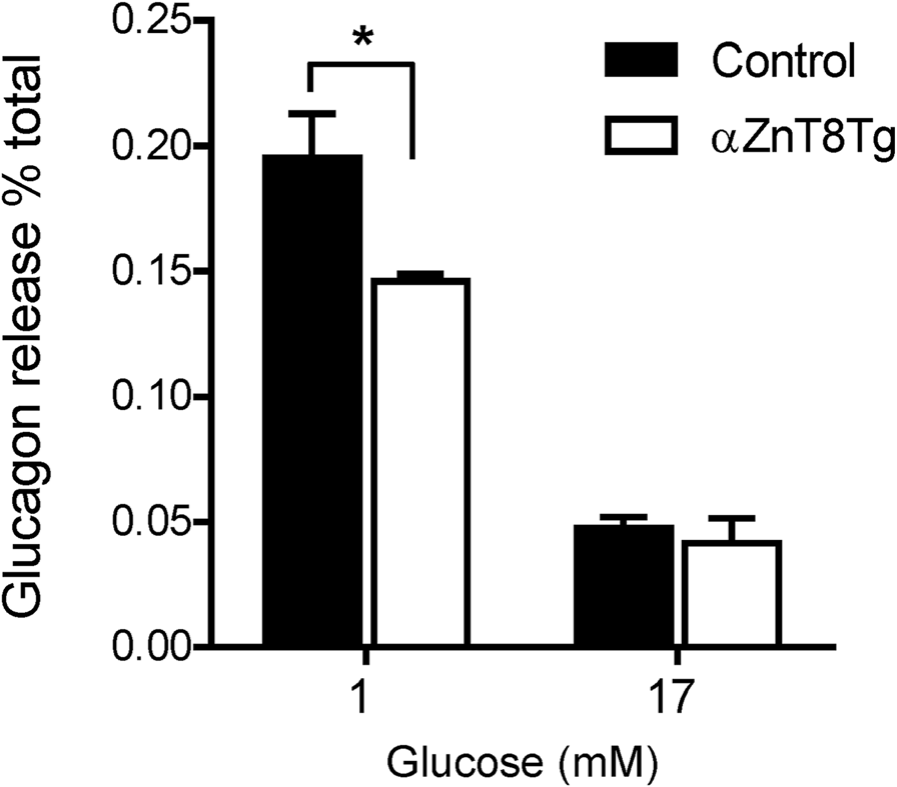
Glucagon secretion from αZnT8Tg islets is impaired in vitro. Isolated islets were incubated at 1 mM or 17 mM glucose for 1 h. Released glucagon was measured using an HTRF assay. Transgenic islets secreted significantly less glucagon compared to control when incubated at 1 mM glucose. Data are mean ± SEM, *n* = 4 separate experiments involving islets from 3 to 4 mice per genotype, *P < 0.05

We sought next to determine whether the impaired glucagon secretion observed in vivo in αZnT8Tg mice chiefly reflected a cell autonomous effect in the α-cell, or a more complex set of changes in the autocrine response to hypoglycaemia, potentially involving multiple tissues (brain, adrenals etc.) [30, 31]. Correspondingly, we observed that when isolated islets were incubated in 1 mM glucose (a concentration in the range which strongly stimulates glucagon release from α-cells) [29], those overexpressing ZnT8 secreted significantly less (~20 %, *p* < 0.05) glucagon that control islets (Fig. 4). At 17 mM glucose the responses did not differ between islets expressing ZnT8 selectively in the α-cell and control islets.

### Impact of ZnT8 over-expression on granular and cytosolic free Zn^2+^ concentrations in clonal α-cells

**Fig. 5:**
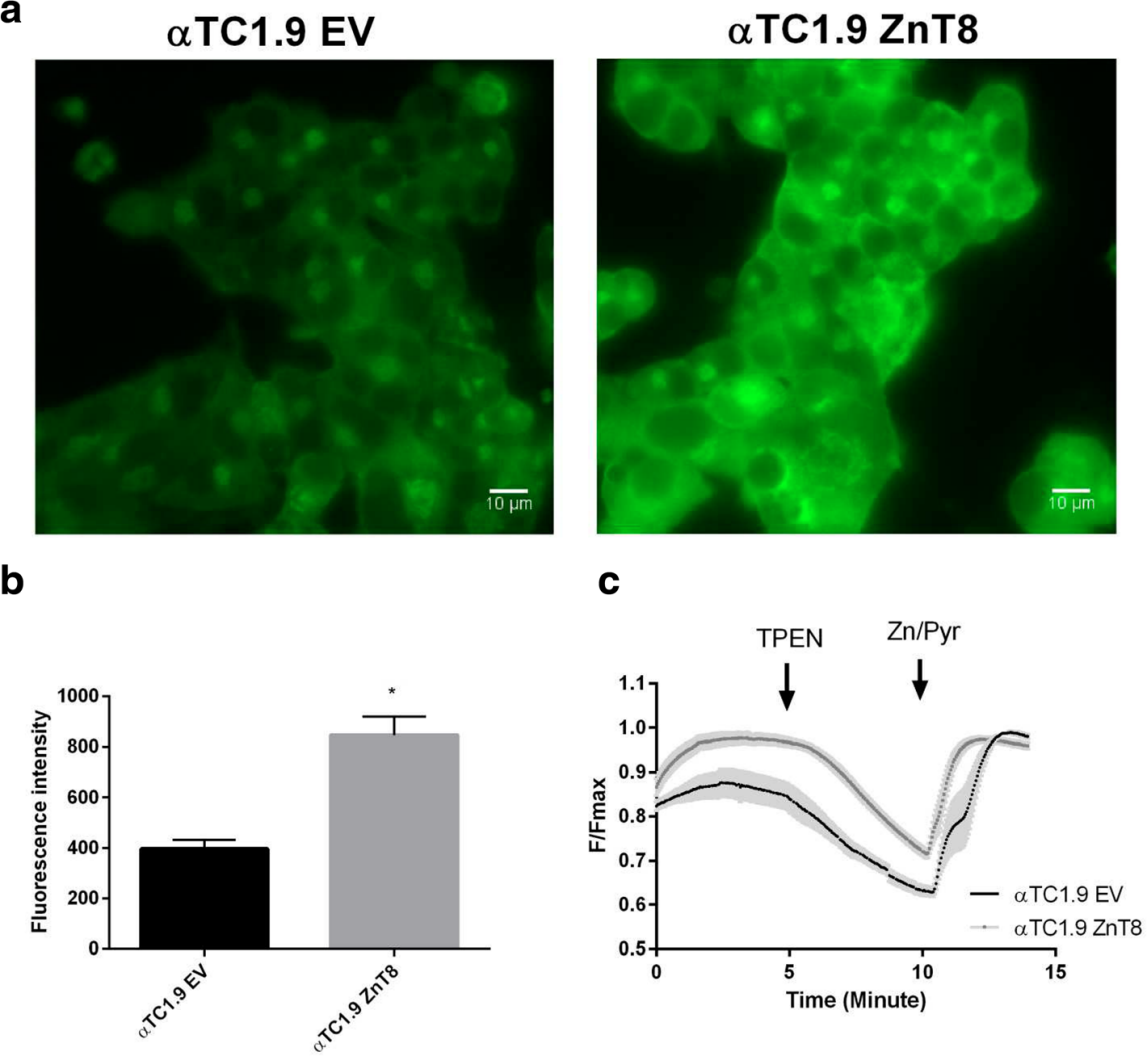
Vesicular free Zn^2+^ assessment in αTC1.9 cells. αTC1.9 cells transfected with an expression vector encoding ZnT8 (αTC1.9 ZnT8) or empty vector (αTC1.9 EV) were incubated with ZinPyr4 (1 μM) for 20 min. before imaging. **a** Representative fields of view for αTC1.9 EV and αTC1.9 ZnT8 cells. **b** ZinPyr4 fluorescence intensity was determined on ten fields of view for two acquisitions, with constant light source power and exposure time. **c** Average traces. Acquisitions were performed under perifusion with imaging buffer and with addition as stated (TPEN 50 μM - pyrithione (5 μM)/Zn^2+^ (100 μM)). Fluorescence intensity ratio was normalised as F/F_max_

**Fig. 6:**
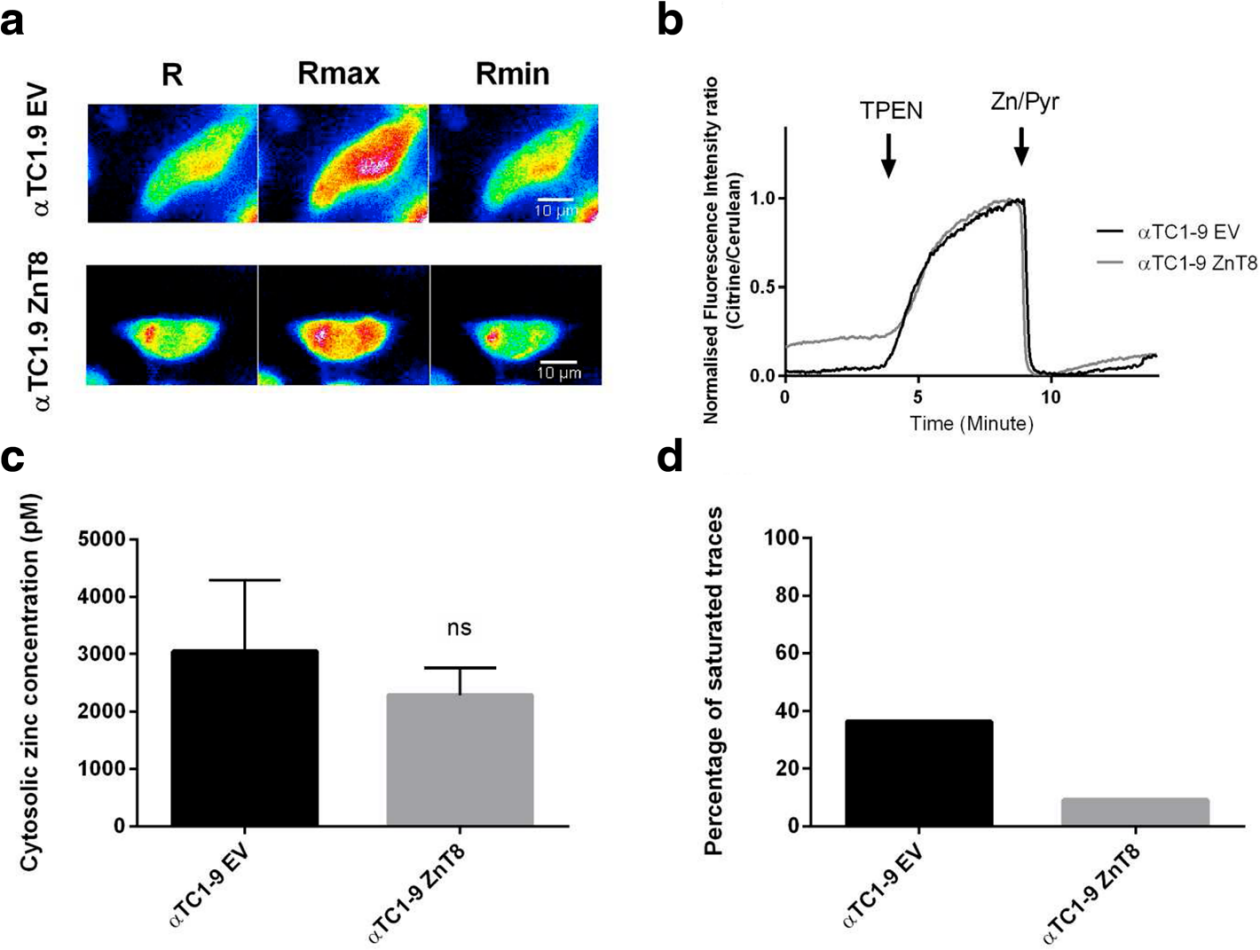
Cytosolic free Zn^2+^ concentration measured using eCALWY-4 in αTC1.9 cells. αTC1.9 cells were co-transfected with an eCALWY-4 construct and with an expression vector for ZnT8 (αTC1.9 ZnT8) or empty vector (αTC1.9 EV). **a** Representative ratiometric images, obtained at steady state (R), under perfusion with the Zn^2+^ chelator N,N,N’,N’-tetrakis (2 pyridylmethyl) ethylenediamine (TPEN) 50 μM (R_max_) and under perfusion with pyrithione (5 μM)/Zn^2+^ (100 μM) (Rmin). **b** Representative traces obtained. Fluorescence intensity ratio was normalised according to the formula: (R-R_min_)/(R_max_-R_min_). **c** Using R, R_min_ and R_max_ values, cytosolic free Zn^2+^ concentrations were calculated for αTC1.9 EV cells (2 experiments - 7 cells) and αTC1.9 ZnT8 cells (2 experiments - 20 cells). **d** Percentage of cells presenting with a saturated sensor response (ie. cytosolic Zn^2+^ concentrations > 8 nM)

In order to determine whether ZnT8 overexpression may act via changes in subcellular free Zn^2+^ concentrations, we monited the latter in secretory granules (Fig. 5) using the granule-accumulated Zn^2+^ probe, Zinpyr4 [17] or in the cytosol with the recombinant-targeted Förster resonance energy-based probe, eCALWY4 [27]. Since individual α cells could not readily be identified in primary mouse islets from the transgenic animals, these experiments were performed using the clonal α-cell line, αTC1-9 [29]. Whereas granular Zn^2+^ was significantly increased in the presence of exogenously-expressed ZnT8, as indicated by a substantial increase in the fluorescence of Zinpyr4 (Fig. 5), free Zn^2+^ concentrations tended to be lowered in the cytosol after ZnT8 over-expression (Fig. 6).

## Discussion

In this report our aim was to overexpress ZnT8 in the pancreatic α-cell in order to investigate the effect on glucagon secretion. The results, which demonstrate interesting parallels with respect to our findings in αZnT8 null animals [17], reveal that overexpression of ZnT8 in the α-cell has no effect on glucose homeostasis or insulin sensitivity, as assessed by intraperitoneal tolerance tests. By contrast, increased α-cell ZnT8 levels strongly affect responses to hypoglycaemia, as studied in hyperinsulinaemic clamps, as well as glucagon secretion in response to low glucose both in vivo and in vitro. The findings from the transgenic mouse with respect to glucagon release are thus reciprocal to those obtained in αZnT8 null mice and further reinforce the view that ZnT8 has an important role in the α-cell in regulating glucagon secretion.

Measured by qPCR in FACS-purified α-cells from αZnT8Tg mice, human ZnT8 mRNA levels were approximately 0.6 times those of β-actin (*Actb*) mRNA (Fig. 1c). This value is similar to that measured for the murine ZnT8 homologue [17]. Assuming similar amplification efficiencies of the primers used for human and rodent ZnT8, and homogeneous expression across the entire α-cell population, the above measurement implies roughly equivalent levels of mouse and human ZnT8 mRNA in transgenic α-cells, i.e. a doubling of the endogenous level in wild type mice. We note, however, that though the more active (W325) human ZnT8 variant was used in the present studies [7], the relative transporter activities of this versus the rodent (Q325) variant are not known, precluding a more accurate estimate of the extent of the increase in ZnT8 activity in αZnT8Tg versus control α-cells.

Likewise, efforts to quantify the degree of human ZnT8 expression in the α-cell at the protein level were unsuccessful. These included immunostaining followed by islet dissociation, FACS analysis, and confocal imaging. Both approaches revealed limited affinity or selectively of the available antibodies towards human ZnT8 (Table 2). Thus, staining of islets did not reveal human ZnT8-positive populations in the face of high background staining (results not shown). Likewise, under FACS analysis, the entire population of islet cells from either control or αZnT8Tg mice was found to be positive for human ZnT8 (results not shown). Similar findings were made over a range of primary and secondary antibody concentrations, and suggest at least partial reactivity of the anti-human antibody towards murine ZnT8. An anti-c-*Myc* antibody, aimed to detect the epitope tag present at the C-terminus of the ZnT8 transgene was also tested. Again, the staining was too weak across a range of primary antibody concentrations for meaningful quantitation (results not shown).

The results here complement those of a similar recent study from our laboratory [23]. In this earlier study, human ZnT8 was over-expressed in the adult β-cell using an insulin promoter-dependent Tet-On system. In the latter model, glucose-induced insulin secretion from βZnT8Tg mice was impaired whereas Zn^2+^ release during stimulated exocytosis was elevated. A similar increase in granular Zn^2+^ content and secretion of these ions might reasonably be expected from αZnT8Tg α-cells especially since targeted deletion of ZnT8 led to a reduction of granular Zn^2+^ levels in both the α [17] and β [23] cell. Likewise, changes in cytosolic free Zn^2+^ cells levels may occur after ZnT8 overexpression (as observed in β cells after the deletion of the transporter) [32]. Measurements using targeted Zn^2+^ probes [33] revealed the expected changes in compartmentalised Zn^2+^ upon ZnT8 over-expression in the α cell line αTC1-9 (Figs. 5 and 6). Thus, it seems likely that ZnT8 over-expression also increased granular Zn^2+^ in the primary α cell in the transgenic mice. Future studies, in which the latter mouse line is crossed to a reporter strain allowing the expression and identification of a fluorescent marker in the α cell [17], will be required to confirm these observations.

In addition to changes in cell function, we do not exclude the possibility that subtle changes (lowering) in α cell mass may also contribute to decreased glucagon release in αZnT8Tg mice, potentiating the impact on the acute release of the hormone observed after islet isolation (Fig. 4). Importantly, such changes may lead to alterations in the activity of signalling pathways the control cell survival [33]. Similarly, changes in the expression of genes involved in glucose sensing by α-cells (e.g. glucokinase, *Gck*, or subunits of ATP-sensitive K^+^ channels, i.e. *Kcnj11*or *Abcc8*, etc.) [34, 35] may also contribute to altered glucagon release from αZnT8Tg mouse islets. We note, however, that alterations in the latter were not observed after ZnT8 ablation [8] or overexpression [23] from β cells. Changes in the expression of genes controlling glucagon synthesis or processing of the prohormone (e.g. *Pcsk1* or *Pcsk2*) would appear unlikely given unaltered total glucagon levels in αZnT8Tg islets (not shown).

Interestingly, over-expression of ZnT8 in the β cell also substantially inhibited glucose-induced insulin release both in vivo and from isolated islets [23]. In both β and α cells this might reflect slower dissolution of crystalline or other higher-order forms of the cargo hormones within secretory granules, impairing the formation and subsequent release of dimers or monomers through a diffusion-limiting pore [36]. βZnT8Tg mice nonetheless displayed enhanced glucose clearance compared to wild-type animals [23]. This change may be the consequence of increased Zn^2+^ release and an insulinomimetic action of these ions on target tissues [33], as well as impaired clearance of insulin by the liver [10]. By contrast, the impaired hypoglycemic response observed in αZnT8Tg mice was largely in line with the observed decrease in plasma glucagon levels, arguing that any increase in Zn^2+^ co-release from the α cell is insufficient to compensate for the lowered levels of the latter hormone. In any case, we note that glucagon and Zn^2+^ are reported to exert opposing effects on glycolysis in the isolated liver cells, whilst exerting a similar, activating effect on glycogen breakdown [37].

## Conclusions

In direct contrast to the activating effect of ZnT8 deletion in α cells [17], overexpression of the transporter in these cells inhibits glucagon release under hypoglycaemic conditions. The present results thus reinforce the view that ZnT8 inhibition may be useful as a means to enhance glucagon release and hypoglycemic responses in the context of T1D. Conversely, activation of ZnT8 in the α-cell, and inhibition of glucagon secretion, may be beneficial in T2D, complementing the effect of ZnT8 overexpression in β cells to enhance insulin action [23]. Our findings also suggest that differences in the impact on T2D risk between rare [12] and more common [2] variants in the human *SLC30A8*/ZnT8 gene [13] might be explained in part by varying actions on glucagon release.

## Abbreviations

FACS, fluorescence-activated cell sorting; GIR, glucose infusion rate; rtTA, reverse tetracyclin transactivator; IPGTT, IPITT, intraperitoneal glucose and insulin tolerance tests, respectively; T1D, T2D, type 1 and type 2 diabetes mellitus, respectively; TMEM, N,N,N’,N’-tetrakis (2 pyridylmethyl) ethylenediamine
